# Aortic Stenosis and the Evolution of Cardiac Damage after Transcatheter Aortic Valve Replacement

**DOI:** 10.3390/jcm13123539

**Published:** 2024-06-17

**Authors:** Fabián Islas, Patrick O’Neill-González, Pilar Jiménez-Quevedo, Luis Nombela-Franco, Sandra Gil-Abizanda, Patricia Mahía-Casado, María Rivadeneira-Ruiz, Eduardo Pozo-Osinalde, Andreina Carbone, Carmen Olmos

**Affiliations:** 1 Instituto Cardiovascular, Hospital Clínico San Carlos, Instituto de Investigación Sanitaria del Hospital Clínico San Carlos (IdISSC), 28040 Madrid, Spain; fabianislas@gmail.com (F.I.); patrickoneillgo@gmail.com (P.O.-G.);; 2 Unidad de Imagen Cardiaca, Hospital Universitario Nuestra Señora del Prado, 45600 Talavera de la Reina, Spain; 3 Cardiology Unit, University of Campania Luigi Vanvitelli, 81100 Naples, Italy

**Keywords:** aortic stenosis, TAVR, cardiac damage staging

## Abstract

**Background/Objectives**: Severe aortic stenosis (AS) is the most frequent valvular heart disease. Models for stratifying cardiac damage associated with aortic stenosis have been developed to predict outcomes following valve replacement. However, evidence regarding morphological and functional evolution, as well as potential changes in the degree of cardiac damage, is limited. We aim to provide information on the evolution of cardiac morphology and the function of patients undergoing transcatheter aortic valve replacement (TAVR) who have been classified using a cardiac damage staging system. **Methods**: In total, 496 patients were included in the analysis, and were classified into four stages based on the extent of cardiac damage as follows: Stage 0, no cardiac damage: left ventricle global longitudinal strain (LV-GLS) < −17%; right ventricular–arterial coupling (RVAc) ≥ 0.35), and absence of significant mitral regurgitation (MR). Stage 1, left-sided subclinical damage: LV-GLS ≥ −17%. Stage 2, left-sided damage: significant MR. Stage 3, right-sided damage: RVAc < 0.35. **Results**: The mean age was 82.1 ± 5.9 years, and 53.0% were female. In total, 24.5% of patients met the criteria for Stage 0, and Stage 1 included 42.8% of patients, Stage 2 included 16.5%, and Stage 3 comprised 16.2% of patients. Mortality was 8.4% for stage 0, 17.4% for stage 1, 25.6% for stage 2, and 28.6% for stage 3 patients (*p* = 0.004). Diabetes mellitus (DM) (*p* = 0.047) and chronic kidney disease (CKD) (*p* = 0.024) were the only clinical predictors of no change or worsening in the stage of cardiac damage. Regarding echocardiographic variables, concomitant tricuspid, and mitral regurgitation, ≥ 2 were both significantly associated with no change or worsening, also (*p* < 0.001). **Conclusions**: Cardiac damage that is secondary to severe aortic stenosis has morphological and functional repercussions that, even after valve replacement, persist and might worsen the prognosis.

## 1. Introduction

Severe aortic stenosis (AS) is the most frequent valvular heart disease in an increasingly older population [1,2]. Different models for stratifying cardiac damage associated with aortic stenosis have been developed [3,4] to predict outcomes following valve replacement, whether surgical or percutaneous. These models have focused on predicting and describing major cardiovascular events, particularly one-year mortality. However, evidence regarding morphological and functional evolution, as well as potential changes in the degree of cardiac damage, is limited. This study aims to provide information on the evolution of the different parameters of cardiac morphology and the function of patients undergoing transcatheter aortic valve replacement (TAVR) who have been classified using a cardiac damage staging system.

## 2. Methods

### 2.1. Design and Setting

This study is a retrospective analysis of prospectively collected data. From 2017 to 2021, all consecutive patients with severe symptomatic AS treated with TAVR in a tertiary care hospital were included in a prospective registry. This study’s protocol was approved by the institutional ethics committee at our hospital, and all patients provided written informed consent.

### 2.2. Definitions, Outcome, and Data Collection

All data were obtained from clinical and imaging records. Data and events of patients were included in a prospective registry, and were subsequently analyzed.

Transthoracic echocardiograms (TTE) before TAVR were performed according to current guidelines [5], and were conducted and/or supervised by experts in cardiovascular imaging. Patients were classified into four stages according to a previously described staging system [6] based on the extent of cardiac damage as follows: Stage 0, no cardiac damage: left ventricle global longitudinal strain (LV-GLS) < −17%; right ventricular-arterial coupling (RVAc) ≥ 0.35, and absence of significant mitral regurgitation (MR). Stage 1, left-sided subclinical damage: LV-GLS ≥ −17%. Stage 2, left-sided damage: significant MR. Stage 3, right-sided damage: RVAc < 0.35.

After one year of follow-up, patients underwent TTE, and they were again classified into the different stages of cardiac damage based on the parameters obtained. In addition, changes in ventricular morphology and function, and their relationship with the stages of cardiac damage, were evaluated.

The primary outcome of this study is the evolution of cardiac damage stage in terms of improvement, no change, or worsening.

### 2.3. Statistical Analysis

Categorical data are presented as frequencies and percentages, and are compared using the χ^2^ test or Fisher’s exact test. Continuous variables were expressed as mean ± standard deviation (SD), and were compared using the Students’ *t*-test and the Mann–Whitney U test as necessary. Assessment for the normality of data was performed using the Shapiro–Wilk test. Logistic regression analysis was used to identify predictors of outcome (change in the stage of cardiac damage). Variables that were statistically significant in the univariable analysis (*p* < 0.05) and which were considered clinically relevant were included in a multivariable logistic regression model. The final model was built based on the Akaike information criterion.

The Kaplan–Meier method was used to calculate the mortality distribution, and curves for cumulative incidence were generated.

All *p*-values were two-sided, and differences were considered to be statistically significant at *p* < 0.05. Statistical analyses were performed with Stata, version 17 (StataCorp, Lakeway Dr. College Station, TX, USA).

## 3. Results

### 3.1. Baseline Characteristics

At baseline, 496 patients were included in the analysis after excluding those with incomplete data and those who underwent valve-in-valve TAVR or any other indication than AS. The mean age was 82.1 ± 5.9 years, and 53.0% were female. In total, 60% of the devices used were balloon-expandable; the type of prosthesis implanted had no relevance to the primary endpoint of the study. After applying our cardiac damage stage system, 24.5% of patients met the criteria for Stage 0, and Stage 1 included 42.8%, Stage 2 included 16.5%, and Stage 3 comprised 16.2% of patients.

### 3.2. Echocardiographic Characteristics

Patients with any degree of cardiac damage showed larger left ventricular end-diastolic volume (LVEDV) (*p* = 0.002), LV mass (*p* < 0.001), and left atrial volume index (LAVI) (*p* < 0.001) compared to those in Stage 0. The E/e’ ratio and pulmonary artery systolic pressure (PSAP) were significantly higher among these patients, also (*p* < 0.001); see Table 1. As for LV ejection fraction (LVEF) and global longitudinal strain (GLS), patients with cardiac damage showed significantly lower values (*p* = <0.001). Significant (≥2) MR was present in 18.9% of patients, and ≥ 2 tricuspid regurgitation (TR) was present in 15.4%.

### 3.3. One-Year Mortality

**Figure 1:**
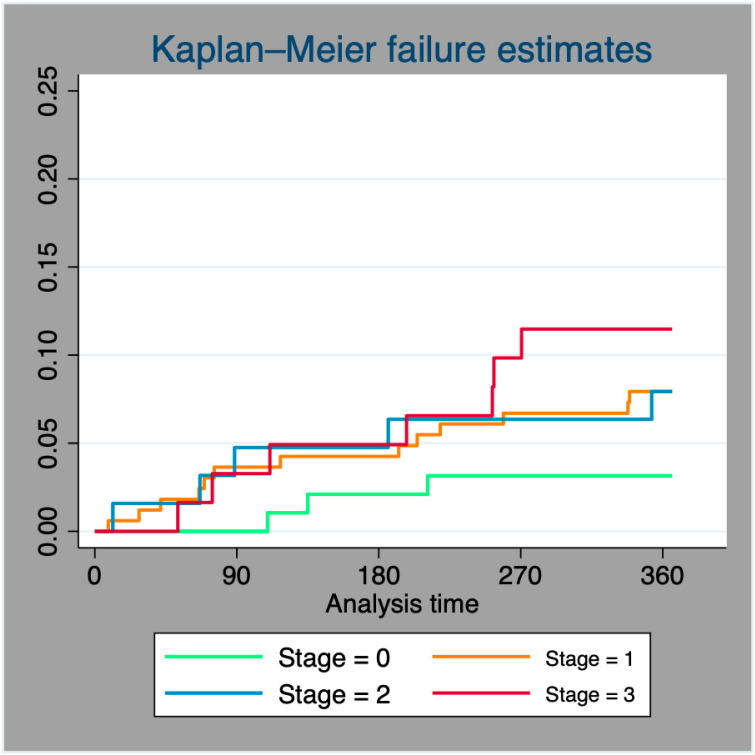
Kaplan–Meier mortality curves.

After one year of follow-up, overall mortality in the cohort was 17.7% (*n* = 88); only 3.3% of deaths were due to CV causes. The main cause of cardiovascular death was heart failure (87.5%, n = 14). The most frequent causes of mortality were infections (36.4%), oncological diseases (19.3%), and COVID-19 infections (12.5%). Mortality showed a directly proportional relationship with the different stages of cardiac damage. It was 8.4% for stage 0, 17.4% for stage 1, 25.6% for stage 2, and 28.6% for stage 3 patients (*p* = 0.004). Kaplan–Meier curves for each staging system show cumulative mortality; see Figure 1.

Clinical variables that showed significant association with overall one-year mortality were chronic kidney disease (CKD) (*p* = <0.001) and Euroscore II, (*p* = 0.039). Hypertension (HTN) and chronic obstructive pulmonary disease (COPD) showed a trend toward significance, *p* = 0.089 and 0.067, respectively.

### 3.4. Evolution of Cardiac Damage at One-Year Follow-Up

**Figure 2:**
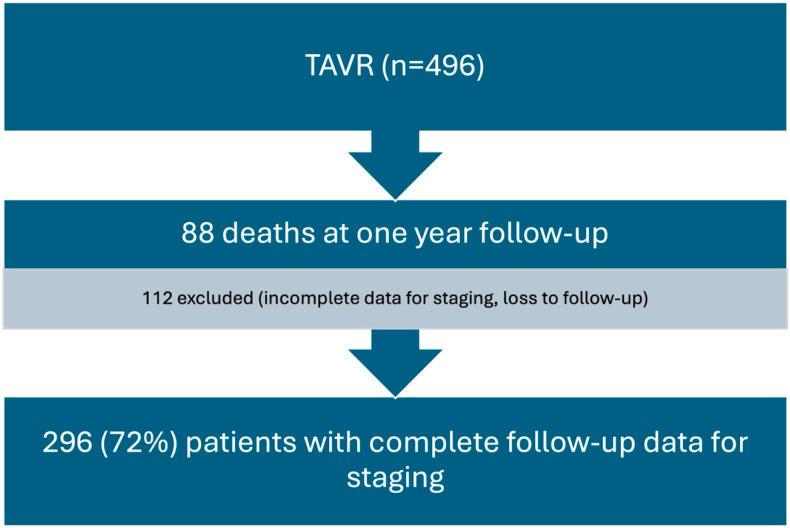
Flow chart of the study population.

After 1 year of follow-up, we obtained data from 296 patients (72%) after excluding those who died or whose echo data were incomplete for cardiac damage classification, mostly GLS; see Figure 2.

**Figure 3:**
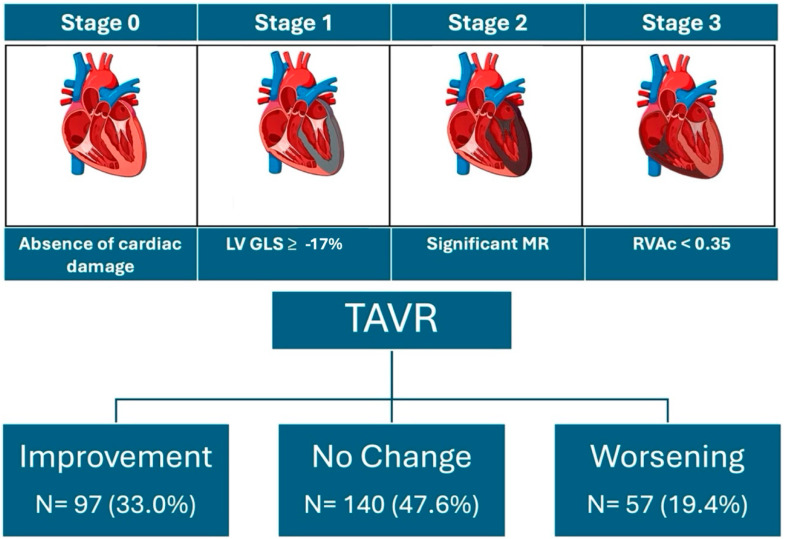
Stages of cardiac damage and changes after one-year follow-up.

**Figure 4:**
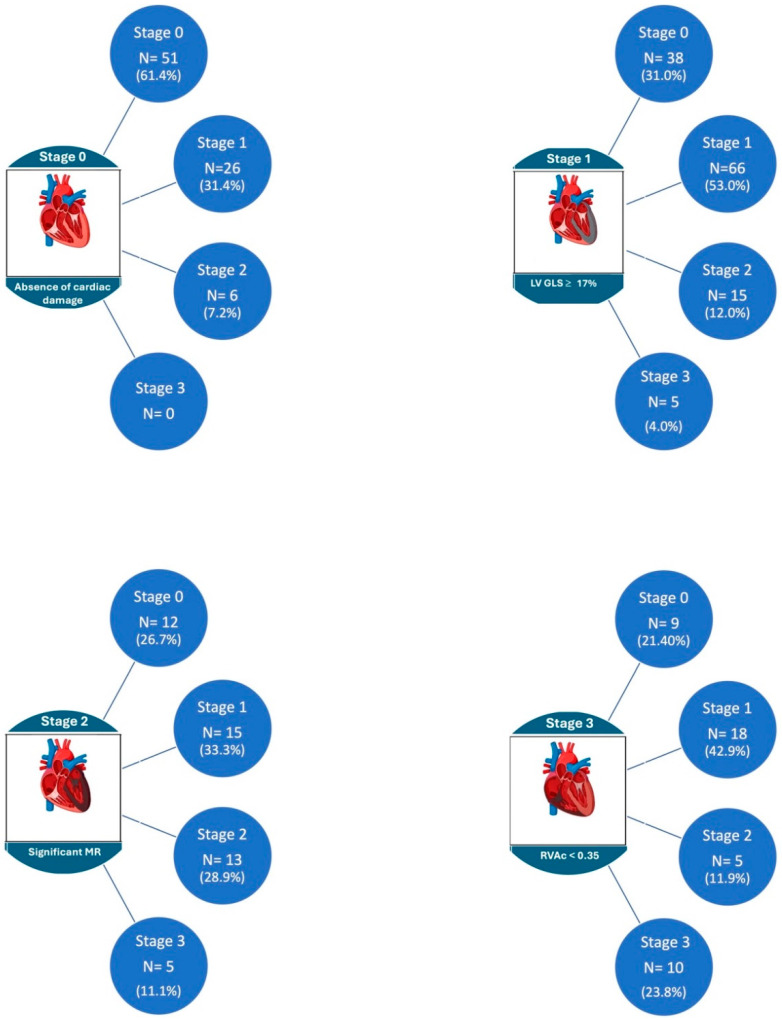
Changes in stages at one-year follow-up.

The changes observed in the stages of cardiac damage after one year are shown in Figure 3 and Figure 4. Of the total sample, 33% of the patients showed improvement in the stage of cardiac damage, 47.6% had no change, and 19.4% showed worsening of their cardiac damage. Among patients in stage 0 at baseline, 62% remained in this category after one year of follow-up, and 38% had a worsening to stages 1 and 2. In stage 1 patients, 69% remained unchanged or worsened; in stage 2, 40% showed no change or worsened; and in stage 3, 24% of patients showed no improvement.

Patients that were classified as having some degree of myocardial damage at baseline persisted with higher LVEDV and LV mass compared to the group without cardiac damage, although the difference was not statistically significant. LAVI was significantly larger (*p* < 0.001), and the E/e’ ratio remained higher in patients with advanced stages of cardiac damage (*p* = 0.284). LVEF and GLS were both lower in patients who showed cardiac damage at follow-up (*p* = 0.005 and *p* < 0.001, respectively); see Table 2.

Regarding post-TAVR ventricular remodeling, patients who showed improvement in the stage of cardiac damage had a greater reduction in LV mass index compared to the unchanged or worsening groups, −14.3 ± 21.5, −10.9 ± 16.3 and −3.9 ± 23.3 gr/m^2^ (*p* = 0.002), respectively. The LVEF had an increase ≥ 10% with relation to baseline in 44.7% of patients who improved the stage of cardiac damage, in 43.5% of those who had no change, and in only 11.8% of patients who showed worsening (*p* = 0.008). Significant MR was present in 14.7% of patients, and showed significant TR in 16.8% of patients.

Interestingly, by grouping the patients into those without cardiac damage or stage 1 (subclinical damage) and those with more advanced stages (2 and 3), we observed that 87.4% of the patients in the first group remained at stage 0 or had improvement, while 37.9% of the patients with more advanced stages did not improve or otherwise worsened (*p* < 0.001).

Diabetes mellitus (DM) (*p* = 0.047) and CKD (*p* = 0.024) were the only clinical variables associated with no change or worsening in the stage of cardiac damage. Regarding echocardiographic variables, concomitant tricuspid and mitral regurgitation ≥ 2 were significantly associated with no change or worsening, also (*p* < 0.001); see Table 3.

## 4. Discussion

The main findings of the present study can be summarized as follows: (1) in our cohort, at baseline, 75% of patients had some degree of cardiac damage, and after one year of follow-up, up to 61% of patients still had some degree of cardiac damage; (2) nearly two thirds of the patients had no change in the stage of cardiac damage or worsened; and (3) patients who underwent TAVR in earlier stages (0–1) showed significantly better outcomes in left ventricular morphology and function, progression of cardiac damage, and mortality.

The pivotal study by Généreux et al. [3], and the subsequent analysis of that series of patients, helps us to understand the importance of timing for the selection of patients undergoing AVR. The use of cardiac damage staging schemes has proven to be of great utility in assessing the prognosis of patients with severe AS undergoing TAVR. However, the results of these studies—including ours—show that a high percentage of patients are intervened when they already have advanced stages of cardiac damage, and many of the parameters included in these stages fail to improve after AVR [3,4,6,7].

Probably one of the main limitations of cardiac damage staging systems is the fact that they attempt to explain morbidity and mortality in patients, who are generally older and have multiple associated pathologies, using exclusively echocardiographic variables. In our cohort, CKD and diabetes were strong predictors of no change or worsening in the stage of cardiac damage; both were very prevalent, and affected 26.5% and 36% of patients, respectively. Recently, Wang et al. [8] reported, in a meta-analysis of 133,624 patients, that all-cause mortality was significantly increased in patients with any degree of CKD, as compared to patients without CKD, both at 30-day, 1-year, and even 2-year follow-up after TAVR. In addition, Gupta et al. [9] described that patients with CKD or end-stage renal disease had a higher incidence of major adverse cardiovascular events (MACE) and pacemaker implantation compared with no CKD patients. Some of the causes attributed to these worse outcomes in patients with CKD are related to age, high EuroSCORE, increased risk of bleeding, acute kidney injury, and exacerbation of pre-existing cardiac problems such as coronary artery disease, heart failure, or conduction disturbances [8,9,10,11].

Furthermore, we observed that diabetes might play a role in patients with no change or worsening in the stage of cardiac damage after 1 year of follow-up. In this regard, Matsumoto et al. [12] described that DM was significantly associated with higher 2-year all-cause mortality; notably, they found that DM was markedly associated with higher mortality, especially in patients with reduced LVEF and high levels of LDL-C. In our study, we found no association between DM and 1-year all-cause mortality. Nevertheless, it has been described that a significant association between diabetes and mortality is found beyond the first year post-TAVR. In the meta-analysis conducted by Abramowitz et al. [13], the main conclusion they obtained is that DM is not a significant incremental short-term risk factor at TAVR, but does confer significant longer-term risk [14]. Thus, adequate follow-up and treatment of DM, as well as all known cardiovascular risk factors, is essential to improve the mid- and long-term prognosis of these patients.

Finally, we would like to highlight the role of concomitant valvular disease on the outcomes of patients undergoing TAVR. In our cohort, both MR and TR were strong predictors of no change or worsening in the stage of cardiac damage. It is known that MR ≥ 2 has an impact on LV morphology and function, not only 1 year after TAVR, but up to 3 years after the procedure, as described in different studies. Muratori et al. [15] reported that an improvement of LV systolic function was observed in all patients, irrespective of etiology and severity of MR. However, a greater degree of positive LV remodeling, as well as a greater degree of MR improvement, was found in moderate-to-severe functional MR. This is particularly important considering that, in our series, 88.6% of MR was of degenerative etiology, which may explain why, in more advanced stages of cardiac damage, the morphological and functional benefits described in that study are not seen in our cohort. The data obtained in different studies regarding etiology point in the same direction; they found similar mortality at 1 year between organic and functional MR, but organic MR was associated with an increased mortality at 3 years of follow-up. Moreover, combining the severity and etiology of MR, the best survival rate was observed in cases with functional MR ≥ 2, and the highest mortality rate was observed in organic MR ≥ 2 [15,16,17].

In our study, TR plays a key role, as it is part of the criteria that determine the stage with the highest risk of mortality (Stage 3 = RVAc < 0.35). Several studies have shown that patients with high-grade TR were more likely to suffer from chronic atrial fibrillation, heart failure with a significantly higher NYHA functional class, worse left and right ventricular function, as well as a higher frequency of right atrial and right ventricular dilatation and pulmonary hypertension [18,19,20]. In addition, the combination of significant TR, pulmonary hypertension (PHT), and RV dysfunction has been described to be a marker of poor prognosis for patients undergoing TAVR [21,22]. Meucci et al. [23], found that post-TAVR RV-PA uncoupling (< 0.55) was independently associated with an increased risk of mortality. Among patients with post-TAVR RV-PA uncoupling, the presence of severe uncoupling (<0.32) identified a subgroup with the worst survival. Even in the absence of significant MR, baseline, or a post-procedure, significant TR and RV dysfunction worsen the prognosis after TAVR [19,23].

Despite the weight of the variables discussed above, we cannot forget other important parameters in the prognosis of these patients, such as GLS, LAVI, or E/e’, among others. The cardiac damage associated with AS is extensive, and requires a detailed evaluation of the entire clinical scenario.

### Limitations

This is a single-center observational study, and therefore has certain limitations. Our sample of patients is relatively small, and at the moment, concerning mortality, we only have the information corresponding to one year of follow-up, so we cannot yet provide information on the prognostic impact of our data in the mid–long term. In this sense, it is in our interest to evaluate the results after 5 years of follow-up, as other series [7] have already reported data after 2 years of follow-up.

## 5. Conclusions

Cardiac damage secondary to severe AS has morphological and functional repercussions that, even after valve replacement, persist and might worsen the prognosis. Despite the existence of various models to determine the stage of cardiac damage and its impact on the outcomes of patients with severe AS, their use is still limited, and, therefore, the benefits of early patient selection are not fully translated into daily practice.

## Figures and Tables

**Table 1:** Baseline clinical and echocardiographic characteristics.

Baseline Stage	Stage 0	Stage 1	Stage 2	Stage 3	*p* Value
**Age, years**	81.8 ± 6.4	81.7 ± 5.8	82.2 ± 5.5	83.3 ± 5.6	0.301
**BMI (kg/m^2^)**	27.9 ± 4.7	28.0 ± 5.9	28.7 ± 7.1	28.0 ± 5.2	0.780
**HTA (%)**	24.0	42.6	17.3	16.0	0.836
**DM (%)**	27.3	41.7	16.6	14.4	0.738
**DLP (%)**	25.4	39.6	17.1	17.9	0.364
**CKD (%)**	17.6	37.2	21.6	23.5	**0.017**
**CAD (%)**	22.6	45.3	16.8	15.3	0.900
**Logistic EuroSCORE**	11.9 ± 7.2	17.5 ± 12.7	19.0 ± 14.9	26.4 ± 15.0	**<0.001**
**LVEF %**	64.1 ± 5.1	55.4 ± 9.4	54.5 ± 13.2	53.1 ± 12.5	**<0.001**
**LVEDV, mL/m^2^**	51.3 ± 13.3	53.3 ± 22.9	58.0 ± 18.9	62.1 ± 24.3	**0.003**
**LVGLS, %**	−17.8 ± 2.8	−15.4 ± 4.2	−15.3 ± 4.6	−15.1 ± 42	**<0.001**
**LV mass, g/m^2^**	116.2 ± 25.0	127.2 ± 28.9	132.6 ± 34.5	140.7 ± 38.9	**<0.001**
**LAVI, mL/m^2^**	42.7 ± 16.3	46.1 ± 16.7	57.5 ± 20.4	66.5 ± 76.6	**<0.001**
**E/e’ ratio**	13.8 ± 4.7	14.7 ± 6.3	17.3 ± 6.1	19.5 ± 6.5	**<0.001**
**PASP, mmHg**	31.0 ± 10.3	31.9 ± 10.7	37.5 ± 12.5	57.2 ± 14.3	**<0.001**
**TAPSE, mm**	22.5 ± 4.2	20.9 ± 4.0	20.6 ± 4.1	15.8 ± 3.8	**<0.001**
**RVAc**	0.81 ± 0.3	0.74 ± 0.3	0.69 ± 0.3	0.28 ± 0.5	**<0.001**

BMI, body mass index; CKD, chronic kidney disease; DLP, dyslipidemia; DM, diabetes mellitus; LAVI, left atrium volume index, LVEDV, left ventricle end-diastolic volume, LVEF left ventricle ejection fraction, LVGLS, left ventricle global longitudinal strain; PASP, pulmonary artery systolic pressure; RVAc, right ventricular-arterial coupling; TAPSE, tricuspid annulus plane systolic excursion.

**Table 2:** Echocardiographic characteristics at one-year follow-up.

Stage 1-Year FU	Stage 0	Stage 1	Stage 2	Stage 3	*p* Value
**LVEF %**	61.4 ± 5.5	58.2 ± 9.3	56.6 ± 9.0	57.7 ± 8.9	**<0.001**
**LVEDV, mL/m^2^**	49.6 ± 14.9	50.8 ± 15.5	52.1 ± 21.1	56.0 ± 21.7	0.223
**LV mass, gr/m^2^**	110.7 ± 31.8	111.4 ± 31.0	113.8 ± 26.6	120.6 ± 26.1	0.307
**LVGLS, %**	−17.8 ± 2.8	−15.4 ± 4.2	−15.3 ± 4.6	−15.1 ± 42	**<0.001**
**LAVI, mL/m^2^**	41.4 ± 16.1	43.9 ± 17.8	55.3 ± 21.7	71.0 ± 74.4	**<0.001**
**E/e’ ratio**	13.8 ± 5.1	14.6 ± 5.5	15.8 ± 5.4	15.9 ± 4.7	0.284
**PASP, mmHg**	26.9 ± 7.4	28.5 ± 11.3	31.5 ± 13.3	37.3 ± 13.6	**<0.001**
**TAPSE, mm**	21.8 ± 4.1	20.4 ± 3.9	20.7 ± 4.6	18.4 ± 4.5	**<0.001**
**RVAc**	0.87 ± 0.3	0.81 ± 0.3	0.77 ± 0.3	0.58 ± 0.3	**<0.001**

LAVI, left atrium volume index; LVEDV, left ventricle end-diastolic volume; LVEF, left ventricle ejection fraction; LVGLS, left ventricle global longitudinal strain; PASP, pulmonary artery systolic pressure; RVAc right ventricular-arterial coupling; TAPSE, tricuspid annulus plane systolic excursion.

**Table 3:** Univariate and multivariate logistic analysis.

	Univariable Logistic Regression Analysis		Multivariable Logistic Regression Analysis	
	Odds Ratio (95% CI)	*p* Value	Odds Ratio (95% CI)	*p* Value
**HTA**	1.49 (0.76–2.93)	0.236		
**DM**	1.55 (1.14–2.17)	**0.014**	1.38 (1.09–2.48)	**0.047**
**DLP**	1.27 (0.74–2.17)	0.374		
**CKD**	2.14 (1.20–3.79)	**0.010**	2.19 (1.15–4.20)	**0.024**
**CAD**	1.58 (0.93–2.67)	0.092		
**EuroSCORE**	1.00 (0.99–1.02)	0.599		
**LVEF**	1.02 (1.00–1.06)	**0.029**		
**GLS**	0.92 (0.86–0.98)	**0.015**		
**MR ≥ 2**	1.83 (1.01–3.32)	**0.047**	3.24 (1.07–9.87)	**<0.001**
**TR ≥ 2**	1.73 (1.36–3.18)	**0.038**	2.33 (1.01–3.99)	**<0.001**

## Data Availability

The datasets used and analysed during the current study are available from the corresponding author upon reasonable request.

## References

[1] Coffey S., Cairns B.J., Iung B. (2016). The modern epidemiology of heart valve disease. Heart.

[2] Vahanian A., Beyersdorf F., Praz F., Milojevic M., Baldus S., Bauersachs J., Capodanno D., Conradi L., De Bonis M., De Paulis R. (2022). 2021 ESC/EACTS Guidelines for the management of valvular heart disease. Eur. Heart J..

[3] Généreux P., Pibarot P., Redfors B., Mack M.J., Makkar R.R., A Jaber W., Svensson L.G., Kapadia S., Tuzcu E.M., Thourani V.H. (2017). Staging classification of aortic stenosis based on the extent of cardiac damage. Eur. Heart J..

[4] Okuno T., Heg D., Lanz J., Praz F., Brugger N., Stortecky S., Windecker S., Pilgrim T. (2021). Refined staging classification of cardiac damage associated with aortic stenosis and outcomes after transcatheter aortic valve implantation. Eur. Heart J. Qual. Care Clin. Outcomes.

[5] Lang R.M., Badano L.P., Mor-Avi V., Afilalo J., Armstrong A., Ernande L., Flachskampf F.A., Foster E., Goldstein S.A., Kuznetsova T. (2015). Recommendations for cardiac chamber quantification by echocardiography in adults: An update from the American Society of Echocardiography and the European Association of Cardiovascular Imaging. J. Am. Soc. Echocardiogr..

[6] Gutierrez-Ortiz E., Olmos C., Carrión-Sanchez I., Jiménez-Quevedo P., Nombela-Franco L., Párraga R., Gil-Abizanda S., Mahía P., Luaces M., de Agustín J.A. (2023). Redefining cardiac damage staging in aortic stenosis: The value of GLS and RVAc. Eur. Heart J. Cardiovasc. Imaging.

[7] Généreux P., Pibarot P., Redfors B., Bax J.J., Zhao Y., Makkar R.R., Kapadia S., Thourani V.H., Mack M.J., Nazif T.M. (2022). Evolution and Prognostic Impact of Cardiac Damage After Aortic Valve Replacement. J. Am. Coll. Cardiol..

[8] Wang J., Liu S., Han X., Chen Y., Chen H., Dong S., Song B. (2022). Impact of Chronic Kidney Disease on the Prognosis of Transcatheter Aortic Valve Replacement in Patients with Aortic Stenosis: A Meta-Analysis of 133624 Patients. Ann. Thorac. Cardiovasc. Surg..

[9] Gupta T., Goel K., Kolte D., Khera S., Villablanca P.A., Aronow W.S., Bortnick A.E., Slovut D.P., Taub C.C., Kizer J.R. (2017). Association of Chronic Kidney Disease With In-Hospital Outcomes of Transcatheter Aortic Valve Replacement. JACC Cardiovasc. Interv..

[10] Allende R., Webb J.G., Munoz-Garcia A.J., de Jaegere P., Tamburino C., Dager A.E., Cheema A., Serra V., Amat-Santos I., Velianou J.L. (2014). Advanced chronic kidney disease in patients undergoing transcatheter aortic valve implantation: Insights on clinical outcomes and prognostic markers from a large cohort of patients. Eur. Heart J..

[11] Gracia E., Wang T.Y., Callahan S., Bilfinger T., Tannous H., Pyo R., Kort S., Skopicki H., Weinstein J., Patel N. (2020). Impact of Severity of Chronic Kidney Disease on Management and Outcomes Following Transcatheter Aortic Valve Replacement With Newer-Generation Transcatheter Valves. J. Invasive Cardiol..

[12] Matsumoto S., Ohno Y., Miyamoto J., Ikari Y., Tada N., Naganuma T., Yamawaki M., Yamanaka F., Shirai S., Mizutani K. (2021). Impact of diabetes mellitus on outcome after transcatheter aortic valve replacement: Identifying high-risk diabetic population from the OCEAN-TAVI registry. Catheter. Cardiovasc. Interv..

[13] Abramowitz Y., Vemulapalli S., Chakravarty T., Li Z., Kapadia S., Holmes D., Matsouaka R.A., Wang A., Cheng W., Forrester J.S. (2017). Clinical Impact of Diabetes Mellitus on Outcomes After Transcatheter Aortic Valve Replacement: Insights From the Society of Thoracic Surgeons/American College of Cardiology Transcatheter Valve Therapy Registry. Circ. Cardiovasc. Interv..

[14] Chorin E., Finkelstein A., Banai S., Aviram G., Barkagan M., Barak L., Keren G., Steinvil A. (2015). Impact of Diabetes Mellitus and Hemoglobin A1C on Outcome After Transcatheter Aortic Valve Implantation. Am. J. Cardiol..

[15] Muratori M., Fusini L., Tamborini G., Ali S.G., Gripari P., Fabbiocchi F., Salvi L., Trabattoni P., Roberto M., Agrifoglio M. (2020). Mitral valve regurgitation in patients undergoing TAVI: Impact of severity and etiology on clinical outcome. Int. J. Cardiol..

[16] Kiramijyan S., Magalhaes M.A., Koifman E., Didier R., Escarcega R.O., Minha S., Baker N.C., Negi S.I., Torguson R., Gai J. (2016). Impact of baseline mitral regurgitation on short- and long-term outcomes following transcatheter aortic valve replacement. Am. Heart J..

[17] Omar S., Aneni E., Escolar E., Mihos C.G., Xydas S., LaPietra A., Beohar N., Arenas I.A. (2020). Tricuspid regurgitation and in-hospital outcomes after transcatheter aortic valve replacement in high-risk patients. J. Thorac. Dis..

[18] Amat-Santos I.J., Castrodeza J., Nombela-Franco L., Muñoz-García A.J., Gutiérrez-Ibanes E., Hernández J.M.d.l.T., Córdoba-Soriano J.G., Jiménez-Quevedo P., Hernández-García J.M., González-Mansilla A. (2018). Tricuspid but not Mitral Regurgitation Determines Mortality After TAVI in Patients With Nonsevere Mitral Regurgitation. Rev. Esp. Cardiol..

[19] Alushi B., Beckhoff F., Leistner D., Franz M., Reinthaler M., Stähli B.E., Morguet A., Figulla H.R., Doenst T., Maisano F. (2019). Pulmonary Hypertension in Patients With Severe Aortic Stenosis: Prognostic Impact After Transcatheter Aortic Valve Replacement: Pulmonary Hypertension in Patients Undergoing TAVR. JACC Cardiovasc. Imaging.

[20] Puehler T., Pommert N.S., Freitag-Wolf S., Seoudy H., Ernst M., Haneya A., Sathananthan J., Sellers S.L., Meier D., Schöttler J. (2024). Tricuspid Regurgitation and TAVR: Outcomes, Risk Factors and Biomarkers. J. Clin. Med..

[21] Ito S., Pislaru S.V., Soo W.M., Huang R., Greason K.L., Mathew V., Sandhu G.S., Eleid M.F., Suri R.M., Oh J.K. (2016). Impact of right ventricular size and function on survival following transcatheter aortic valve replacement. Int. J. Cardiol..

[22] Grevious S.N., Fernandes M.F., Annor A.K., Ibrahim M., Croix G.R.S., de Marchena E., Cohen M.G., Alfonso C.E. (2020). Prognostic Assessment of Right Ventricular Systolic Dysfunction on Post-Transcatheter Aortic Valve Replacement Short-Term Outcomes: Systematic Review and Meta-Analysis. J. Am. Heart Assoc..

[23] Fan J., Liu X., Yu L., Sun Y., Jaiswal S., Zhu Q., Chen H., He Y., Wang L., Ren K. (2019). Impact of tricuspid regurgitation and right ventricular dysfunction on outcomes after transcatheter aortic valve replacement: A systematic review and meta-analysis. Clin. Cardiol..

